# Ethnic inequalities in mental health and socioeconomic status among older women living with HIV: results from the PRIME Study

**DOI:** 10.1136/sextrans-2020-054788

**Published:** 2021-03-29

**Authors:** Danielle Solomon, Shema Tariq, Jon Alldis, Fiona Burns, Richard Gilson, Caroline Sabin, Lorraine Sherr, Fiona Pettit, Rageshri Dhairyawan

**Affiliations:** 1 Institute for Global Health, University College London, London, UK; 2 London South Bank University, London, UK; 3 Royal Free London NHS Foundation Trust, London, UK; 4 UK Community Advisory Board, London, UK; 5 Department of Infection and Immunity, Barts Health NHS Trust, London, UK

**Keywords:** HIV, women, ethnic groups, race factors

## Abstract

**Objectives:**

Women living with HIV in the UK are an ethnically diverse group with significant psychosocial challenges. Increasing numbers are reaching older age. We describe psychological and socioeconomic factors among women with HIV in England aged 45–60 and explore associations with ethnicity.

**Methods:**

Analysis of cross-sectional data on 724 women recruited to the PRIME Study. Psychological symptoms were measured using the Patient Health Questionnaire 4 and social isolation with a modified Duke-UNC Functional Social Support Scale.

**Results:**

Black African (BA) women were more likely than Black Caribbean or White British (WB) women to have a university education (48.3%, 27.0%, 25.7%, respectively, p<0.001), but were not more likely to be employed (68.4%, 61.4%, 65.2%, p=0.56) and were less likely to have enough money to meet their basic needs (56.4%, 63.0%, 82.9%, p<0.001). BA women were less likely to report being diagnosed with depression than WB women (adjusted odds ratio (aOR) 0.40, p<0.001) but more likely to report current psychological distress (aOR 3.34, p<0.05).

**Conclusions:**

We report high levels of poverty, psychological distress and social isolation in this ethnically diverse group of midlife women with HIV, especially among those who were BA. Despite being more likely to experience psychological distress, BA women were less likely to have been diagnosed with depression suggesting a possible inequity in access to mental health services. Holistic HIV care requires awareness of the psychosocial needs of older women living with HIV, which may be more pronounced in racially minoritised communities, and prompt referral for support including psychology, peer support and advice about benefits.

## Introduction

The COVID-19 pandemic and the Black Lives Matter movement have exposed the consequences of continuing and widespread structural racism. Describing and understanding health inequalities among people from ethnic minority groups has now become a matter of urgency.

The majority of women living with HIV in the UK are from racially minoritised communities; two-thirds are of Black African (BA) or Black Caribbean (BC) origin. Due to advances in HIV treatment and care, people living with HIV are living longer; in 2018, 14 923 women attending HIV care were aged 45 years or over with 11 115 women of potentially menopausal age (45–56 years), a fivefold increase over 10 years.^1^

A study^2^ of 170 women living with HIV in London, comparing those aged under 45 years with those 45 or over, found that the older group was significantly less likely to be in a relationship and had higher levels of anxiety, depression and other psychological symptoms. However, despite increasing recognition of the importance of quality of life, there is a paucity of research exploring psychosocial well-being among older women living with HIV.^2^

The burden of poor mental health among people living with HIV, and its impact on clinical outcomes, is well recognised.^3 4^ However, despite well documented ethnic inequalities in mental health and access to mental healthcare in the general population,^5^ we lack robust data on the association between racially minoritised status and mental health among people living with HIV in the UK, especially women. Without these data, we are unable to identify and address disparities in health and well-being in this key population.

Using data from the PRIME (Positive TRansItions through the Menopause) Study, we investigate the association between ethnicity and (i) socioeconomic factors and (ii) mental health among women living with HIV aged 45–60 years.

## Methods

### The PRIME Study

The PRIME study was a mixed-methods observational study exploring the impact of the menopause on the health and well-being of women living with HIV. The study recruited 869 women living with HIV aged 45–60 attending one of 21 National Health Service HIV clinics in England between February 2016 and June 2017. Methods are described in detail elsewhere.^6^

### Variables

Demographic factors (including ethnicity) were ascertained via self-report. CD4 count and HIV viral load were obtained from clinical records, and missing values were completed via self-report. Current psychological distress was measured by the Patient Health Questionnaire 4 screening tool,^7^ scores ranging from 0 to 12, with a score ≥6 indicating psychological distress. Social isolation was measured using a validated modified version of the Duke-UNC Functional Support Scale,^8^ scores ranging from 5 to 25. A score ≥12 was classified as having poor social support.

### Statistical analysis

In order to explore associations between ethnicity and psychosocial factors, we restricted the sample to women reporting their ethnicity as White British, BA or BC (n=724).

First, we investigated the relationship between ethnicity and a range of sociodemographic and lifestyle factors. These sociodemographic factors were chosen as they had all previously been found to be associated with mental health outcomes. We started by using multivariable logistic regression models to calculate adjusted odds ratios (aORs) for the association between ethnicity and employment. To identify potential confounders, we performed χ^2^ tests or Kruskal-Wallis tests (as appropriate) to assess the relationship between ethnicity, employment and a range of potentially confounding variables. Variables that were associated (p<0.2) with both ethnicity and employment were included in the multivariable model. Similar methods were then used to investigate the relationship between ethnicity and education, income, social isolation, high risk alcohol use, smoking and recreational drug use. We then examined the relationship between ethnicity and self-reported history of diagnosed depression and current psychological distress.

Analyses were performed using Stata 15 (Stata. 2017. Stata Statistical Software: Release 15. College Station, Texas, USA).

### Theoretical framework

The PRIME study is informed theoretically by the concept of *intersectionality*, an analytic framework that seeks to understand how multiple social categories combine and intersect to shape experience and disadvantage.^9^ In this analysis, we explore how gender, age, ethnicity and HIV status intersect to impact women’s lives.

## Results

Despite nearly two-thirds of women (64.7%) having an educational background equivalent to ‘A-level’ or above, nearly a third were currently unemployed, and 40% reported not having enough money to meet their basic needs either most or all of the time. Nearly 40% of women scored above the cut-off for social isolation. Nearly a third had ever been diagnosed with depression, and 28.9% were currently on antidepressants. Approximately one in four women (27.1%) scored above the cut-off for current psychological distress (table 1).

**Table 1:** Characteristics of the sample, stratified by ethnic group*

	Black African	White British	Black Caribbean	P value†
	n=607 (71.6%)	n=71 (8.4%)	n=46 (5.4%)	
Median age (years)	49	51	50	0.37
Employment				0.56
Employed	397 (65.4)	45 (63.4)	27 (58.7)	
Unemployed	183 (30.1)	24 (33.8)	17 (37.0)	
Education				<0.001
Did not complete school	57 (9.4)	12 (16.9)	9 (19.6)	
‘O’ level‡	120 (19.8)	26 (36.6)	15 (32.6)	
‘A’ level§	118 (19.4)	14 (19.7)	3 (6.5)	
University	275 (45.3)	18 (25.4)	10 (21.7)	
Enough money for basic needs				
All/most the time	337 (56.4)	58 (82.9)	29 (63.0)	<0.001
Some/none of the time	260 (43.6)	12 (17.1)	17 (37.0)	
Born in the UK				
Yes	14 (2.3)	67 (95.7)	20 (45.5)	<0.001
No	584 (97.7)	3 (4.3)	24 (54.6)	
Immigration status				
Secure	527	64	39	<0.001
Insecure	60	0	2	
Social isolation				
No	242 (39.9)	18 (25.4)	22 (47.8)	0.03
Yes (score ≥12)	365 (60.1)	53 (74.7)	24 (52.2)	
Current smoker				
No	576 (97.5)	48 (69.6)	34 (77.3)	<0.001
Yes	15 (2.5)	21 (30.4)	10 (22.7)	
High risk alcohol use				
No	39 (6.9)	50 (75.8)	35 (87.5)	<0.001
Yes (AUDIT ≥5)	529 (93.1)	16 (24.2)	5 (12.5)	
Recreational drug use				
No	589 (98.8)	60 (89.6)	40 (93.0)	<0.001
Yes	7 (1.2)	7 (10.4)	3 (7.0)	
Ever diagnosed with depression				
Yes	156 (26.5)	37 (52.9)	21 (45.7)	<0.001
No	433 (73.5)	33 (47.1)	25 (54.4)	
Currently using antidepressants (if ever diagnosed with depression)				
Yes	68 (26.8)	19 (40.4)	17 (70.8)	0.166
No	186 (73.2)	28 (59.6)	7 (29.2)	
Psychological distress				
Yes (PHQ-4 ≥6)	139 (25.0)	11 (15.7)	14 (34.2)	0.08
No	417 (75.0)	59 (84.3)	27 (65.8)	
Years since diagnosis (IQR)	14 (10–17)	13 (6–23)	10 (5–14)	0.009
Most recent CD4 count (cells/mm^3^)				
≥500	358 (66.8)	48 (68.6)	20 (66.7)	0.96
200–499	142 (26.5)	19 (27.1)	8 (26.7)	
<200	36 (6.72)	3 (4.3)	2 (6.7)	
Most recent HIV viral load				
Undetectable	498 (87.5)	64 (90.1)	35 (92.1)	0.60
Detectable	71 (12.5)	7 (9.9)	3 (7.9)	

*Number of respondents varies between questions due to missing data.

†χ^2^ or Kruskal-Wallis test.

‡Equivalent to completing US Grade 10.

§Equivalent to completing US Grade 12.

PHQ4, Patient Health Questionnaire 4.

Of the 721 women included in this analysis, the majority (71.6%) were BA. On univariable analysis, ethnicity was associated with education, employment and having enough money to meet basic needs. A far larger proportion of BA women (48.3%) had a university education compared with White British (25.7%) and BC women (27.0%, p<0.001) (table 1). However, when compared with White British women, there was no evidence of a difference in the odds of employment for either BA (aOR 0.71 (95% CI 0.41 to 1.40)) or BC (0.75 (0.30 to 1.89)) women. The odds of having enough money to meet basic needs were significantly lower for both BA (0.14 (0.06 to 0.34)) and BC (0.20 (0.07 to 0.57)) women, than in those of White British ethnicity.

Ethnicity was also associated with social isolation, diagnosed depression and current psychological distress. BC and BA women were more likely to be socially isolated than White British women (2.68 (1.12 to 6.38) and 1.95 (1.01 to 3.67), respectively). Furthermore, the odds of current psychological distress were significantly higher among BC (4.81 (1.61 to 14.41)) and BA (3.34 (1.38 to 8.13)) than among White British women. Despite this, BA women had lower odds of ever having been diagnosed with depression (0.40 (0.22 to 0.71)) than White British women, with no difference between women of BC and White British ethnicity (0.80 (0.35 to 1.86)).

## Discussion

In the largest study to date of psychosocial factors in older women living with HIV in England, we report a high prevalence of poverty, social isolation and poor mental health, all of which were worse among those from racially-minoritised groups. BA women were most likely to be university educated, when compared with BC and White British women, but this did not equate to higher levels of employment. Along with BC women, BA women were also less likely to have enough money to meet their basic needs. This is consistent with data in the general population,^10^ and data from a study of people living with HIV in 2004–2005,^11^ showing that being Black or from a racially minoritised community is associated with greater socioeconomic hardship, a consequence of structural racism. BC and BA women were *more* likely to be socially isolated and experience psychological distress, but *less* likely than White British women to be diagnosed with depression or to be on antidepressants. This reflects data in the general population demonstrating significant ethnic disparities in access to mental healthcare and treatment, despite higher rates of mental health diagnoses among those from ethnic minority groups.^5^ Recognised barriers to care include mental health-related stigma, distrust of services, poor cultural competency, racial discrimination, a lack of recognition of symptoms by the patient and clinician, inadequate referral and a reliance on community solutions such as faith groups.^12^ The majority of our sample was born outside of the UK, and it has been shown that migration itself can be a psychosocial stressor.^12^

The cross-sectional nature of our study limits our ability to infer causality, and the findings cannot be extrapolated to women younger or older than our inclusion range of 45–60 years. As we recruited from HIV clinics, we had a sampling bias, only collecting data from women who attend clinic. Finally, this was a secondary analysis of existing data; we also lack data on HIV stigma, racism and income, which are key variables when looking at health disparities in HIV.

This work builds on the previous scant literature on ethnic disparities in psychosocial well-being in people living with HIV in the UK. Our analysis was informed by intersectionality theory, allowing us to foreground the impact of age, ethnicity, gender and HIV status on women’s health and well-being.^9^ Our sample is representative of the UK population of women living with HIV, where most women are engaged in HIV care, experiencing virological suppression on treatment and are of BA ethnicity.^6^

Professionals working with older women living with HIV should be aware of the prevalence of psychosocial stressors in this group, particularly among Black and ethnic minority women who live with the consequences of structural racism. Furthermore, assessment and management of their needs must be proactive, holistic and intersectional in its approach. Our findings reinforce national recommendations that people living with HIV should be screened yearly and at potential trigger points for symptoms of mental illness.^13^ Treating mental illness has been shown to improve HIV outcomes such as adherence,^14^ and we recommend that clinics have access to psychological support that is culturally competent including psychiatry and psychology. Although HIV-related stigma was not measured in this study, it is associated with depression, social isolation and decreased use of health and social care services.^15^ We strongly recommend that peer support is offered to women living with HIV to try and address this. We also recommend that women are regularly asked about financial hardship and social isolation, and that clinics have referral pathways to organisations that can advise on issues such as benefits, housing and immigration, and that these organisations have continued funding in order to provide this support.

Since June 2020, the global Black Lives Matter movement and the COVID-19 pandemic have forced society to recognise the pervasive and pernicious effects of racism. This includes structural racism, which is a driver of persistent and longstanding health inequalities in a wide range of health conditions. It is therefore imperative that we establish an evidence base on ethnic disparities in health and well-being among people living with HIV, using an intersectional approach, in order to address inequalities effectively.
